# The Eyes Cannot See What the Mind Does Not Know: Autochthonous Dengue in South Florida

**DOI:** 10.1016/j.acepjo.2024.100003

**Published:** 2025-01-09

**Authors:** Sabrina Ginsburg, Elizabeth May-Smith, Erin Smith, Christopher Freeman

**Affiliations:** Department of Emergency Medicine, Jackson Memorial Hospital, University of Miami, Miami, Florida, USA

**Keywords:** dengue fever, Aedes aegypti, Aedes albopictus, flavivirus, febrile illness, travel history, autochthonous

## Abstract

Dengue is a mosquito-borne arbovirus that, when it infects humans, can cause a range of illnesses from asymptomatic infection to febrile illness and myalgias that, in rare cases, can be severe or fatal. Dengue has historically been considered a tropical disease in the United States, acquired only by international travelers. However, the incidence of locally transmitted (autochthonous) dengue in nontravelers is increasing in the United States, likely because of the expansion of the range of *Aedes aegypti* and *A. albopictus*, the virus’s mosquito vector. Our case report describes a patient in Florida who was infected with dengue and had no international travel history. As the number of locally transmitted dengue cases is expected to rise, the diagnosis and management of dengue will be of increased importance to emergency medicine physicians in the United States.

## 1 Introduction

Dengue virus is a ribonucleic acid (RNA) flavivirus, one of many viral genera that are considered arboviruses or arthropod-borne viruses. Dengue, like other arboviruses (Zika, chikungunya, and yellow fever), is spread by mosquitoes from the *Aedes* family, the *A. aegypti* and *A. albopictus*.^1^ Dengue is typically considered in patients with a febrile illness who have traveled internationally within the past 10 to 14 days. However, there have been an increasing number of locally transmitted (autochthonous) dengue cases in the United States (U.S.).^2^^,^^3^ According to the Centers for Disease Control and Prevention, 1219 cases of locally acquired dengue were reported in the U.S. in 2023 alone, including 192 cases in the continental U.S.^2^ Dengue can be asymptomatic in up to 50% of infections and can have nonspecific and varied presentations in symptomatic patients, making diagnosis challenging.^1^ Treatment of dengue is largely symptomatic, with careful fluid resuscitation at the forefront of management.^1^^,^^3^ Most cases are self-limiting. However, it is essential for the emergency medicine physician to make a timely diagnosis and initiate supportive treatment to prevent severe complications in the small number of patients that might enter the critical phase of illness.

## 2 Case

The patient was a previously healthy 28-year-old woman who presented to the emergency department (ED) with a chief complaint of fever for 6 days. She reported daily intermittent fevers ranging from 38 to 40 °C accompanied by moderate to severe headache, retrobulbar pain, bilateral neck pain, back pain, nausea, and vomiting. She denied any sick contacts and recent local or international travel in the past 6 months. The patient’s review of symptoms was negative for respiratory illness, abdominal pain, chest pain, or shortness of breath. Initial vital signs were a temperature of 37.1 °C, heart rate of 72 beats per minute, blood pressure of 96/61 mm Hg, respiratory rate of 20 breaths per minute, and oxygen saturation of 98% on room air. On examination, the patient was uncomfortable and mildly ill-appearing. Her head examination was significant for photophobia, with a normal ocular examination. No oropharyngeal erythema, exudate, or petechiae was noted. Her neck examination was significant for no lymphadenopathy or meningismus with a negative Kernig and Brudzinski sign. The neurologic examination was normal; the patient was alert and oriented ×3, cranial nerves 2 to 12 were intact, strength was 5/5 in upper and lower extremities, and sensation was intact to light touch in all 4 extremities. Her gait was normal with negative Romberg. Her abdominal examination was normal, without tenderness or hepatosplenomegaly. The skin examination was negative for rashes, bruising, or petechiae. Her capillary refill was <2 seconds, and her skin was warm and well-perfused. Her remaining examination was normal.

During her course in the ED, the patient received intravenous fluids, ondansetron, acetaminophen, and methocarbamol for her symptoms with moderate improvement. Blood work revealed leukopenia with a white blood cell count of 2800/μL, normal platelets at 157/μL, hemoglobin at 14.6 g/dL, and aspartate transaminase and alanine transaminase of 73 U/L and 56 U/L, respectively. The extended viral respiratory panel was negative. There was no evidence of urinary tract infection, and the urinary pregnancy test was negative. Mild transaminitis, leukopenia, and borderline thrombocytopenia in the setting of headache, retrobulbar pain, severe myalgias, and viral symptoms raised concern for viral vector-borne illness; therefore, chikungunya and dengue polymerase chain reaction (PCR) were sent, and we performed a tourniquet test that was negative. Though the patient did not have a travel history, Miami’s proximity to the Caribbean and prior cases of endemic dengue in Florida and the Southeastern U.S. prompted evaluation for mosquito-borne illness. Noncontrast computed tomography scan of the brain was negative for acute intracranial abnormalities. After reviewing diagnostic results with the patient, we discussed the risks vs benefits of performing a lumbar puncture to assess for viral meningitis. The patient eventually declined and was discharged home with strict return precautions.

The dengue blood serum PCR for virus 1 or 3 was positive 4 days later. The patient was contacted regarding the result. She reported that she was afebrile with improvement in body aches but had some persistent lethargy. The patient confirmed no prior history of dengue infection. At the follow-up 1 week later, the patient had improved significantly with resolution of her symptoms.

## 3 Discussion

Dengue virus is becoming an increasingly common infection in the U.S., both in travelers and nontravelers alike. With symptoms resembling other viral infections like chikungunya, Zika, and even influenza, promptly identifying dengue in the ED can be challenging. Dengue has the following 3 distinct phases: febrile, critical, and recovery.^1^^,^^3^ The febrile phase consists of a flu-like illness with nonspecific clues to suggest dengue, such as leukopenia, thrombocytopenia, transaminitis, positive tourniquet test, severe myalgias, and headaches.^1^ The tourniquet test is a procedure that determines capillary fragility by inflating a blood pressure cuff around the upper arm to the point between systolic and diastolic blood pressure for 5 minutes. The air in the cuff is then released, and the number of petechiae below the antecubital fossa is counted after 2 minutes. Greater than 10 petechiae within 1 square inch on the skin indicates a positive test result.^4^ The tourniquet test is typically only used in resource-limited settings as it has minimal diagnostic accuracy with a reported sensitivity of 58% and specificity of 71% but is immediately available to physicians at the bedside.^4^ In endemic areas, the World Health Organization makes a presumptive diagnosis of dengue based on fever and 2 of the following criteria: anorexia and nausea, rash, aches and pain, leukopenia, and positive tourniquet test. For most patients, dengue is self-limited, but a small percentage of patients progress to the critical phase.^1^^,^^3^

The critical phase is mediated by the host’s immune response and inflammatory mediators, leading to tissue destruction and increased membrane permeability.^5^ Clinically, the critical phase is characterized by defervescence, the appearance of warning signs including abdominal pain or tenderness, persistent vomiting, liver enlargement, fluid accumulation, mucosal bleeding, lethargy or restlessness, and an increase in hematocrit level with a decrease in platelet levels. In some patients, rapid progression to severe dengue can lead to shock, hemorrhage, end-organ damage, and even death.^1^^,^^3^ Although no specific treatment is available for dengue, intense clinical monitoring along with supportive care can significantly lower mortality rates.^6^

The World Health Organization has developed treatment recommendations for patients based on their clinical manifestations that categorize them into groups A to C, but these groups do not correlate directly with the dengue phases of illness.^7^ Group A patients are those with presumed or confirmed dengue without warning signs of severe illness who are able to tolerate fluid ingestion and are urinating appropriately. Warning signs of severe dengue include abdominal pain or tenderness, persistent vomiting, fluid accumulation, mucosal bleeding, lethargy or restlessness, liver enlargement > 2 cm, and increasing hematocrit with decreasing platelet count. Patients that are placed in group A can be discharged with return precautions, monitoring for any warning signs or increasing severity of illness.^7^

Patients in groups B and C should be admitted and managed as inpatients. Patients in group B are those with presumed or confirmed dengue with warning signs or coexisting conditions that could complicate dengue management (obesity, pregnancy, old age, infancy, diabetes, and renal failure) and poor social support. Patients in group C with presumed or confirmed dengue that are in the critical phase of the disease as defined by shock, severe organ dysfunction, bleeding, or plasma leakage that can lead to third spacing and fluid accumulation require emergency stabilization and intensive care unit-level care to treat their organ dysfunction and shock.^7^

In the ED, timely diagnosis of a patient’s condition is imperative, along with appropriate risk stratification. In Florida in 2019, there was a locally acquired case of dengue that resulted in patient fatality because of a delay in diagnosis and, subsequently, appropriate fluid resuscitation and diagnosis-specific care.^8^ When a patient presents to the ED with nonspecific symptoms of fever and malaise, the differential diagnosis list needs to remain broad to allow for optimal patient management. “The eyes cannot see what the mind doesn't know” is a common medical aphorism. Although influenza and other common viruses may be higher on the differential, it is crucial, now more than ever, for an ED clinician in Southern states not to succumb to availability bias and to consider the diagnosis when the clinical picture supports it. Ordering the appropriate work-up that takes into consideration additional diagnoses despite their rarity in the U.S., such as dengue virus, malaria, chikungunya virus, and Zika virus, is critical. If we do not consider these viruses, we will not diagnose the disease. PCR tests and viral antigen testing to definitively diagnose dengue virus should become part of the standard hospital laboratory inventory, especially in the Southeastern U.S., where the *Aedes* mosquito population is predicted to grow.^9^ The true prevalence of autochthonous dengue is unknown because of the limited availability and utilization of testing in this population; if we do not test for it, we will not find it. This being said, using a shotgun approach to test for all possible viruses may not always be an appropriate use of resources. It is a delicate balance in which physicians must contemplate testing selectively using their clinical judgment.

Dengue is typically found in tropical and subtropical regions, notably Central and South America, the Caribbean, Asia, and Africa. Almost 50% of the human population lives in at-risk areas for dengue virus.^10^ Cases of dengue virus within North America have historically occurred in patients who have recently traveled to these endemic areas.^1^ However, within the past decade, there have been documented cases in Florida and Texas of patients acquiring dengue virus via *A. aegypti* and *A. albopictus* with no history of recent travel, suggesting that the mosquito may be migrating.^11^ Recent data modeling climate change and urbanization indicate that the Southeastern U.S. is becoming increasingly habitable for the *Aedes* mosquito and, thus, a suitable environment for the native spread of arbovirus infections such as dengue.^11^ The increasing climate will allow for the continued spread of the *Aedes* mosquito, posing a substantial risk to human health in these newly endemic locations.^12^

In conclusion, although the incidence of dengue fever in the U.S. remains relatively low at present, there is a risk that it may soon become endemic in the South.^11^ It is imperative that ED providers become increasingly aware of the possibility of encountering a patient with dengue fever with no history of recent international travel. In patients where there is clinical concern of dengue, it is essential to appropriately evaluate for any warning signs of severe dengue and admit patients at risk for decompensation for further management and care.

## Funding and Support

By *JACEP Open* policy, all authors are required to disclose any and all commercial, financial, and other relationships in any way related to the subject of this article as per ICMJE conflict of interest guidelines (see www.icmje.org). The authors have stated that no such relationships exist.

## Conflict of Interest

All authors have affirmed they have no conflicts of interest to declare.
